# Poor prognosis of male triple-positive breast Cancer patients: a propensity score matched SEER analysis and molecular portraits

**DOI:** 10.1186/s12885-021-08267-9

**Published:** 2021-05-08

**Authors:** Biyuan Wang, Hui Wang, Andi Zhao, Mi Zhang, Jin Yang

**Affiliations:** Department of Medical Oncology, First Affiliated Hospital of Xi’an Jiaotong University, 277 Yanta West Road, Xi’an, 710061 Shaanxi Province China

**Keywords:** Breast cancer prognosis, Breast cancer subtype, Male breast cancer, Propensity score matched analysis, Genomic features

## Abstract

**Background:**

The purpose of this study was to explore clinicalpathology features, molecular features and outcome of male breast cancer patients who expressed ER, PR as well as HER-2, namely triple-positive male breast cancer (TP-MBC), and compared them with triple-positive female breast cancer patients (TP-FBC).

**Methods:**

TP-MBC and TP-FBC from 2010 to 2017 were selected from the Surveillance, Epidemiology, and End Results database (SEER). Kaplan-Meier plotter and multivariable Cox regression model were applied to analyse the difference between TP-MBC and TP-FBC on cancer-specific survival (CSS) and overall survival (OS). Propensity score matched (PSM) analysis was used to ensure well-balanced characteristics. 7 cases TP-MBC and 174 cases TP-FBC patients with the genomic and clinical information were identified from the cohort of The Cancer Genome Atlas (TCGA) and the Memorial Sloan Kettering (MSK).

**Result:**

336 TP-MBC and 33,339 TP-FBC patients were taken into the study. The percentages of TP-MBC in MBC patients were higher than the rates of TP-FBC in FBC patients from 2010 to 2017 except 2012. Compared with TP-FBC, more TP-MBC were staged III (17.9% vs. 13.5%) or stage IV (11.0% vs. 6.9%). TP-MBC were more frequently to be older than 65-years-old (47.0% vs. 29.3%), Balck (15.2% vs. 10.8%), ductal carcinoma (91.7% vs. 84.4%) and metastases to lung (4.5% vs. 2.1%) or bone (8.6% vs. 4.7%). TP-MBC had worse OS and CSS than TP-FBC in all stages (*P* < 0.001). In multivariable prediction model of TPBC, male patients had a higher risk than female. Lastly, the worse OS (*P* < 0.001) and CSS (*P* = 0.013) were seen in the 1:3 PSM analysis between TP-MBC and TP-FBC. Genomic analysis revealed that TP-MBCs have some notable rare mutations, like ERBB2, ERBB3, RB1, CDK12, FGFR2, IDH1, AGO2, GATA3, and some of them are not discovered in TP-FBC.

**Conclusion:**

TP-MBC had a worse survival than TP-FBC, and there were different genomic features between two groups. Current knowledge and treatment to TP-MBC maybe inadequate and remain to be explored.

**Supplementary Information:**

The online version contains supplementary material available at 10.1186/s12885-021-08267-9.

## Background

As one of the most common cancers, breast cancer accounts for 15% of all new cancer diagnoses in the United states [1]. Most of these cases are female breast cancer (FBC) while male breast cancer (MBC) comprised about 1% [1, 2]. The incidence of MBC has continually increased [3, 4] although it is a relatively rare type of breast cancer, and studies on MBC were limited and insufficient so far.

For decades, oncologists and researchers have had an in-depth study of different clinical characteristics, molecular features, treatment response and prognosis among subtypes in breast cancer. As the markers of hormone therapy, patients with the positive expression of progesterone receptor (PR) and/or estrogen receptor (ER) are usually classified into luminal subtype and given endocrine therapy. MBC is associated with elders, more Black patients, larger tumor, less lobular carcinoma and higher percentage of hormone receptor (HR)-positive than women [2–5]. On the other hand, human epidermal growth factor receptor (HER-2), another important biomarker, is the dominant driver in cancers when over-expressed. About one quarter of FBC are HER-2 positive, and half of them also express HR. According to population-based studies about MBC, the percentages of ER+ MBC are reported to be about 90% or more than 95% [6, 7]. The percentages of PR + MBC ranged from 60 to 90%, generally to be about 80.0% [7–9]. The rates of HER2 positive among MBC differ from the testing methods and the different region. By using IHC to assess expression of HER2 protein, the rates are often less than 10% [10–12]. The percentages were increased when methods like FISH/CISH are applied to define Her2 amplification [13, 14]. MBC in India, Turkish showed high HER2 positive rates (> 20%) [15, 16]. Generally, treatment to male patients is similar to famale patients, except aromatase inhibitor alone is not recommended [5, 10].

Studies which focused on survival and prognosis of MBC are quite small and contradictory when compared with large-population based studies of FBC. The relative survival rate of MBC has been found to be similar to FBC in some research [3, 5, 17], but worse than FBC in other reports [12, 18–20]. Race, lymph node involvement, tumor size, androgen receptor, tumor grade and age at diagnose seem to be prognostic factors in MBC [3, 18, 21]. HR+ MBC patients, especially early-stage patients, have worse survival than HR + FBC patients. For HR + and HER2 + patients, the better survival in FBC was not exact [22]. According to these studies, the outcome of MBC is still difficult to draw clear conclusions based on molecular subtype.

Recent studies showed that the subset of ER/PR/HER-2 positive, namely “triple-positive breast cancer (TPBC)”, represents a unique and complicated entity. Co-expression of HRs and HER-2 means the activation of both pathways, and the cross-talk between two pathways at the same time [23–25]. The expression of HRs in HER-2 positive cancers is often at a lower level than HER-2 negative cancers. Low level of HRs may represent the poor response to endocrine therapy. The resistance to HER-2 blockade was also found in TPBC disease [23]. Thus the exploration on this subtype has representative significance in MBC. As a distinct subset of HER-2 positive breast cancer, there is no data of the survival and prognosis in triple positive male breast cancer (TP-MBC) as well as outcome compared with triple positive female breast cancer (TP-FBC).

Based on the above, a more detailed analysis is needed to report the distinct features and survival of TP-MBC. Here, we use the TP-FBC which diagnosed at the same time as control to investigate the clinical characteristics and outcomes of TP-MBC relative to TP-FBC. Specially, PSM analysis is used to reduce confounding bias between male and female groups. Genomic landscape showed the difference between TP-MBC and TP-FBC. This study is based on the data using patient data from the Surveillance, Epidemiology, and End Results database of individuals diagnosed between 2010 and 2017, as well as the somatic mutation data from TCGA and MSK cohorts.

## Materials and methods

### Patient selection

Data used in this study was from the SEER database, which is openly accessible and freely available for researchers. We used the SEER*Stat software (version 8.3.6) with a data user agreement. A total of 491,913 patients diagnosed with breast cancer between 2010 to 2017 were included. The data for patients’ age, T stage, N stage, M stage, grade, race, surgery status, histological types, ER, PR, HER-2 status and metastasis status were identified. Triple positive breast cancer is defined as the breast cancer patients with positive codes in three SEER variables: ER Status Recode Breast Cancer (1990+), PR Status Recode Breast Cancer (1990+), Derived HER2 Recode (2010+).We identified a total of 33,675 patients with triple positive receptors and further classified these TPBC patients into 336 male patients and 33,339 female patients.

We used two cohorts——TCGA and MSK to examine the somatic mutation of TPBC. Totally 7 cases TP-MBC and 174 cases TP-FBC were identified according to ER, PR status and HER2 immunohistochemistry (IHC) and/or fluorescence in situ hybridization (FISH) results. Information of the somatic mutation and clinical features (including gender, DFS, histology and testing method of HER2) were downloaded and collected from CbioPortal (https://www.cbioportal.org/datasets) at the same time. The 618 gene list identified in MSK-IMPACT & Foundation One CDx were used to compare difference between TP-MBC and TP-FBC. The R packages GenVisR (https://bioconductor.org/packages/GenVisR/) were used to analyse the mutated genes of TPBC.

### Statistical analysis

The baseline characteristics of patients with different sexes were estimated by chi-square test. Kaplan-Meier survival analyses were used to assess the long-term CSS and OS between TP-MBC and TP-FBC. Multivariate Cox regression models were applied to analyze the influence of the risk factors on survival of TPBC patients. We also compared OS and CSS of TP-MBC and TP-FBC in stage-matched subgroups using Kaplan-Meier survival curves and log rank tests. The R software (version × 64 3.5.1, http://www.r-project.org) was used to construct the PSM analysis with 1:3 matching method to reduce confounding bias.

Cancer specific survival was calculated from the date of diagnosis to the date of cancer-related death, or the date that patient was last known to be alive. All data was analyzed using SPSS 22.0 (SPSS Inc. Chicago, IL, USA). All tests were two-tailed, and statistical significance was set at *P* < 0.05.

## Results

### Clinicopathological characteristics of patients

**Fig. 1:**
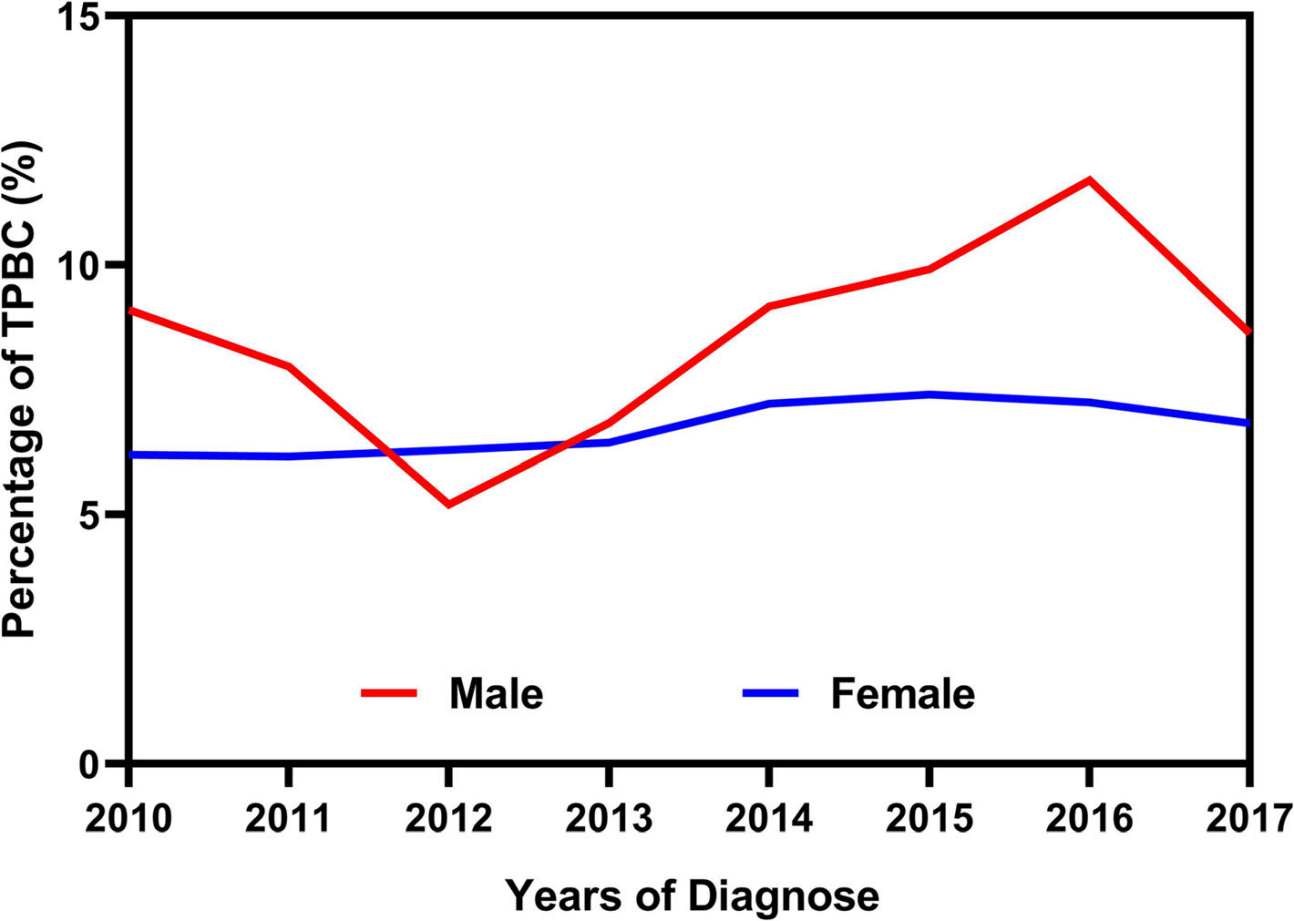
The percentage of TP-FBC/TP-MBC to total FBC/MBC from 2010 to 2017

Among 491,913 patients originally identified from SEER database, cases of 33,339 TP-FBCs and 336 TP-MBCs from 2010 to 2017 were included in our study. According to the percentage of TP-FBC/TP-MBC to total FBC/MBC at each year (range from 5.2 to 11.7%), we firstly showed the trends of the subsets in 8 years (Fig. 1). Generally, the subtype of TPBC was more prevalent in males than that in females with the exception of 2012.

Clinical pathological characteristics of TP-MBC compared with TP-FBC were summarized in Table 1. TP-MBC patients are significantly older than TP-FBC (*P* < 0.001), patients older than 65 years account for almost half in TP-MBC. TP-MBC had less Asian/pacific islanders (5.3% vs. 10.5%, *P* < 0.001), more ductal carcinoma (91.7% vs. 84.4%, *P* < 0.001), higher clinical stage (stage III 17.9% vs. 13.5%; *P* < 0.001) as well as T stage (T4: 13.7% vs. 5.7%; *P* < 0.001), N stage (N3: 6.0% vs. 3.8%; *P* < 0.001), M stage (M1: 11.0% vs.6.9%; *P* < 0.001). However, there was no significant difference in tumor grade and surgery status between TP-MBC and TP-FBC.

**Table 1:** Clinical pathological characteristics of TP-MBC compared with TP-FBC

	TP-MBC (*n* = 336)	TP-FBC (*n* = 33,339)	*P*-value
Age			< 0.001
≤ 50	46 (13.7)	10,928 (32.8)	
50< ≤65	132 (39.3)	12,628 (37.9)	
> 65	158 (47.0)	9783 (29.3)	
Race			< 0.001
White	266 (79.2)	25,738 (77.2)	
Black	51 (15.2)	3588 (10.8)	
A/PI	18 (5.3)	3525 (10.5)	
Other	1 (0.3)	488 (1.5)	
Histology			< 0.001
Ductal	308 (91.7)	28,165 (84.4)	
Lobular and Mix	13 (3.8)	4053 (12.2)	
Other	15 (4.5)	1121 (3.4)	
Grade			0.122
I-II	149 (44.3)	16,565 (49.7)	
III-IV	171 (50.9)	15,112 (45.3)	
Unknown	16 (4.8)	1662 (5.0)	
Stage			< 0.001
I	79 (23.5)	13,068 (39.2)	
II	147 (43.7)	12,606 (37.8)	
III	60 (17.9)	4477 (13.5)	
IV	37 (11.0)	2313 (6.9)	
Unknown	13 (3.9)	875 (2.6)	
T			< 0.001
T0–1	106 (31.5)	16,075 (48.2)	
T2	160 (47.6)	11,926 (35.8)	
T3	11 (3.3)	2344 (7.0)	
T4	46 (13.7)	1894 (5.7)	
Unknown	13 (3.9)	1100 (3.3)	
N			< 0.001
N0	160 (47.6)	19,891 (59.7)	
N1	118 (35.1)	9436 (28.3)	
N2	29 (8.6)	2120 (6.4)	
N3	20 (6.0)	1289 (3.8)	
unknown	9 (2.7)	603 (1.8)	
M			0.006
M0	297 (88.4)	30,942 (92.8)	
M1	37 (11.0)	2311 (6.9)	
Unknown	2 (0.6)	86 (0.3)	
Surgery			0.083
Yes	285 (84.8)	29,494 (88.5)	
No	45 (13.4)	3254 (9.8)	
Unknown	6 (1.8)	591 (1.8)	

In Table 2, data of distant organ metastasis in TP-MBC and TP-FBC was shown. Compared with TP-FBC, TP-MBC patients had higher proportions of bone metastasis (8.6% vs. 4.7%; *P* < 0.001) and lung metastasis (4.5% vs. 2.1%; *P* < 0.001). Significant difference was not found in the brain metastasis or liver metastasis between TP-MBC and TP-FBC.

**Table 2:** Comparison of distant organ metastasis patterns in TP-MBC and TP-FBC

		TP-MBC (*n* = 336)	TP-FBC (*n* = 33,339)	*P*-value
Bone Metastases	+	29 (8.6)	1556 (4.7)	0.001
	–	307 (91.4)	31,783 (95.3)	
Lung Metastases	+	15 (4.5)	699 (2.1)	0.007
	–	321 (95.5)	32,640 (97.9)	
Liver Metastases	+	5 (1.5)	739 (2.2)	0.366
	–	331 (98.5)	32,600 (97.8)	
Brain Metastases	+	2 (0.6)	168 (0.5)	0.814
	–	334 (99.4)	33,171 (99.5)	

### Outcome of TP-MBC compared with TP-FBC

**Fig. 2:**
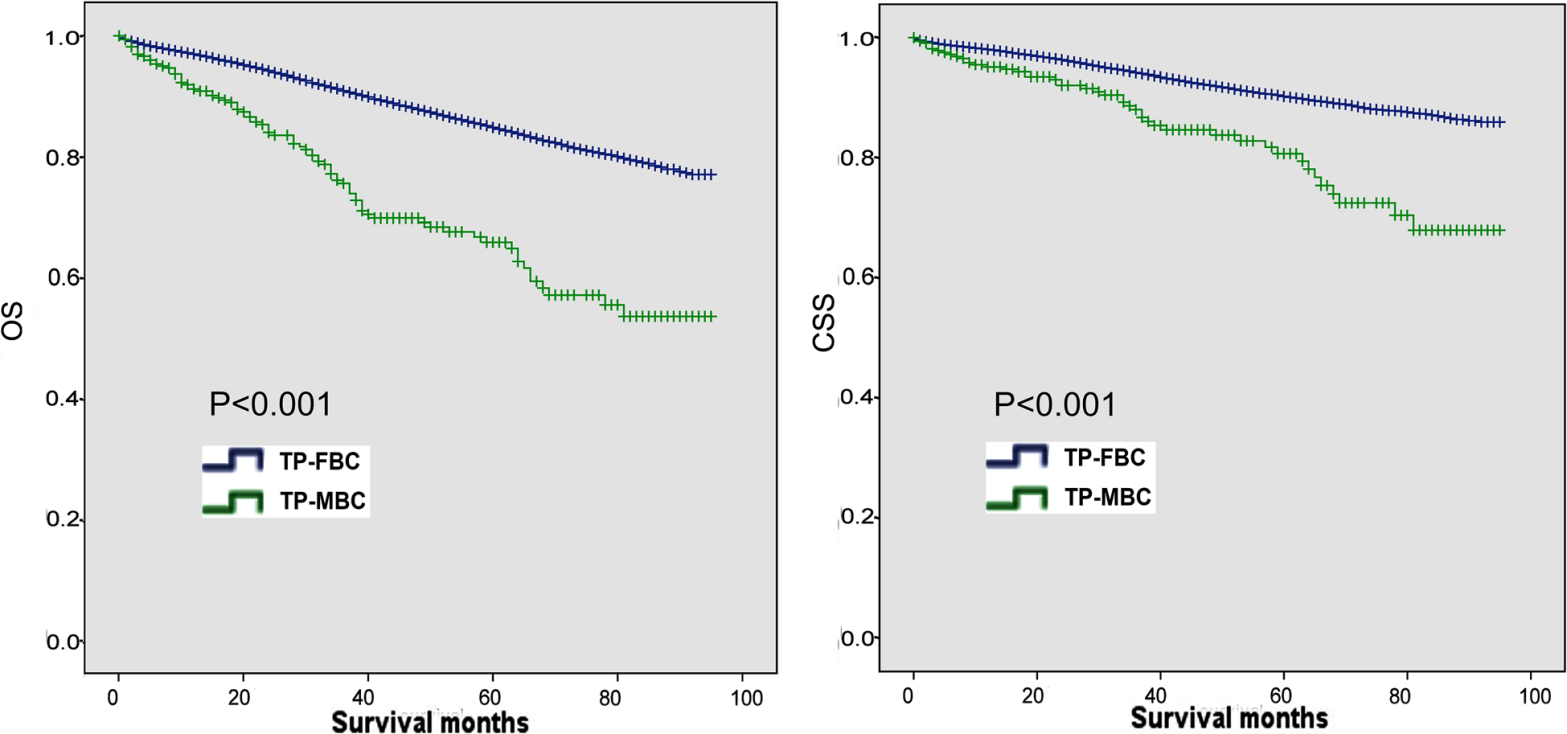
Kaplan–Meier analyses of OS and CSS in TP-MBC and TP-FBC

**Fig. 3:**
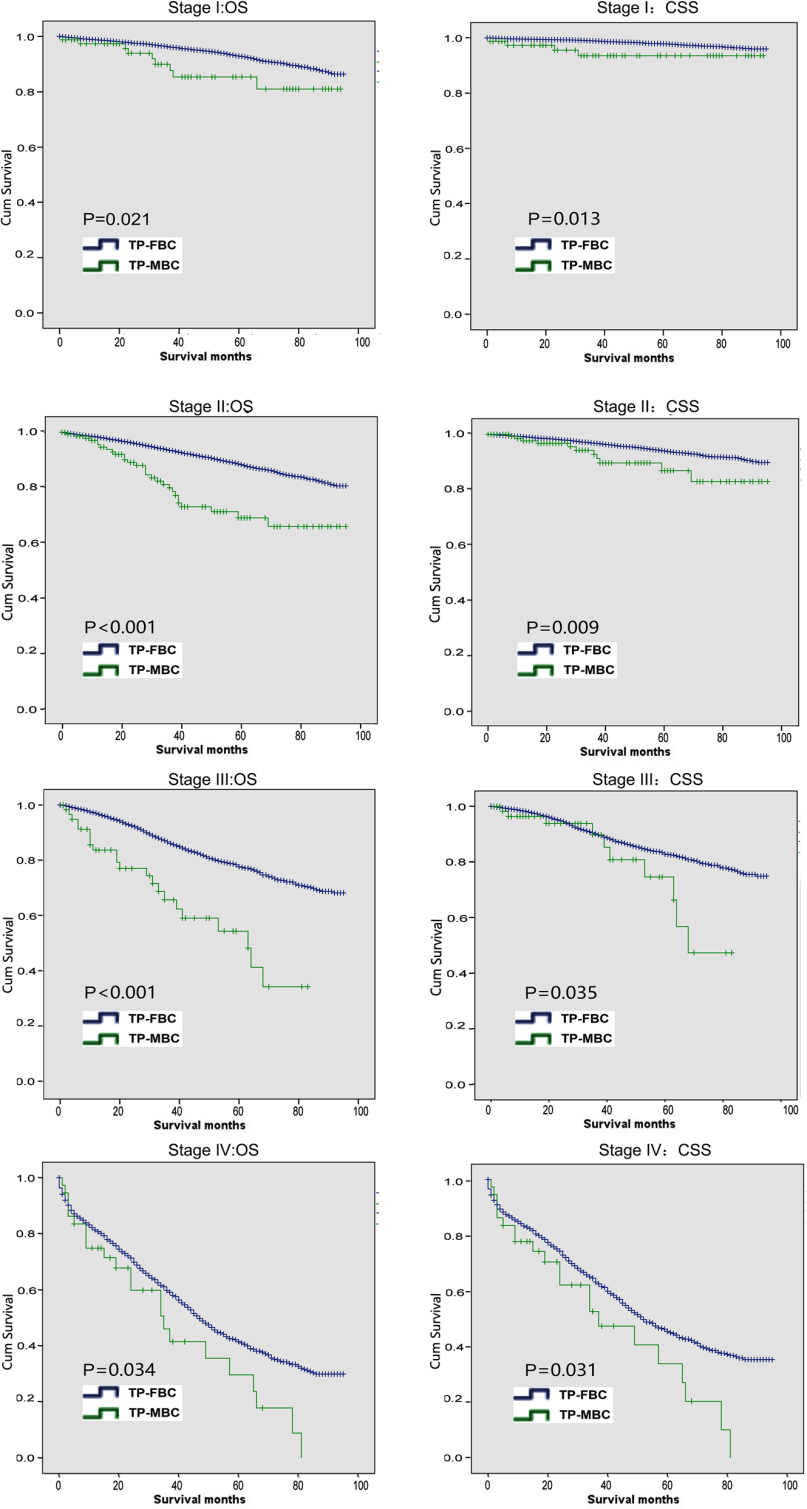
Comparison of OS and CSS in different stages of TP-MBC and TP-FBC

Kaplan–Meier analysis showed that there were significant differences of OS and CSS between TP-MBC and TP-FBC (Fig. 2). Moreover, we performed stratified survival analysis according to the clinical stage. As shown in Fig. 3, prognosis of TP-FBC were better than that of TP-MBC in stage-stratified survival analysis.

In univariate analysis and multivariate analysis of TPBC (Table 3), male patients have worse OS/CSS than female patients (OS:*P* < 0.001; CSS:*P* = 0.002). Sex remained to be an independent risk factor in TPBC patients, when taking age, race, stage, histology, and tumor grade into multi-cox model.

**Table 3:** Univariate and multivariate survival analyses of TPBC

	OS				CSS			
	Univariate analysis ***P***-value	Multivariate analysis			Univariate analysis ***P***-value	Multivariate analysis		
		HR	95%CI	***P***-value		HR	95%CI	***P***-value
**Age**	< 0.001				< 0.001			
≤50		reference				reference		
50< ≤65		1.820	1.634–2.026	< 0.001		1.499	1.329–1.690	< 0.001
> 65		5.233	4.734–5.784	< 0.001		3.045	2.709–3.423	< 0.001
**Race**	< 0.001				< 0.001			
White		reference				reference		
Black		1.227	1.110–1.355	< 0.001		1.277	1.130–1.443	< 0.001
A/PI		0.715	0.621–0.822	< 0.001		0.751	0.632–0.893	0.001
**Histology**	< 0.001				< 0.001			
Ductal		reference				reference		
Lobular and Mix		0.886	0.796–0.986	0.027		0.956	0.831–1.099	0.528
Other		1.194	1.002–1.422	0.047		1.011	0.795–1.285	0.931
**Grade**	< 0.001				< 0.001			
I-II		reference				reference		
III-IV		1.160	1.082–1.244	< 0.001		1.365	1.246–1.495	< 0.001
**T**	< 0.001				< 0.001			
T0–1		reference				reference		
T2		1.635	1.497–1.787	< 0.001		2.123	1.863–2.419	< 0.001
T3		1.906	1.666–2.181	< 0.001		2.838	2.392–3.368	< 0.001
T4		2.386	2.107–2.702	< 0.001		3.388	2.878–3.989	< 0.001
Unknown		1.461	1.225–1.742	< 0.001		2.367	1.906–2.939	< 0.001
**N**	< 0.001				< 0.001			
N0		reference				reference		
N1		1.153	1.059–1.257	0.001		1.440	1.285–1.615	< 0.001
N2		1.643	1.450–1.862	< 0.001		2.241	1.922–2.614	< 0.001
N3		1.905	1.669–2.175	< 0.001		2.434	2.075–2.854	< 0.001
Unknown		1.653	1.387–1.969	< 0.001		1.804	1.449–2.245	< 0.001
**M**	< 0.001				< 0.001			
M0		reference				reference		
M1		2.061	1.866–2.277	< 0.001		3.945	3.425–4.543	< 0.001
**Surgery**	< 0.001				< 0.001			
Yes		reference				reference		
No		4.014	3.640–4.425	< 0.001		4.22253	3.748–4.757	< 0.001
Unknown		1.666	1.262–2.198	< 0.001		1.984	1.438–2.737	< 0.001
**Sex**	< 0.001				< 0.001			
Female		reference				reference		
Male		1.827	1.467–2.275	< 0.001		1.603	1.190–2.159	0.002

### PSM analysis

**Fig. 4:**
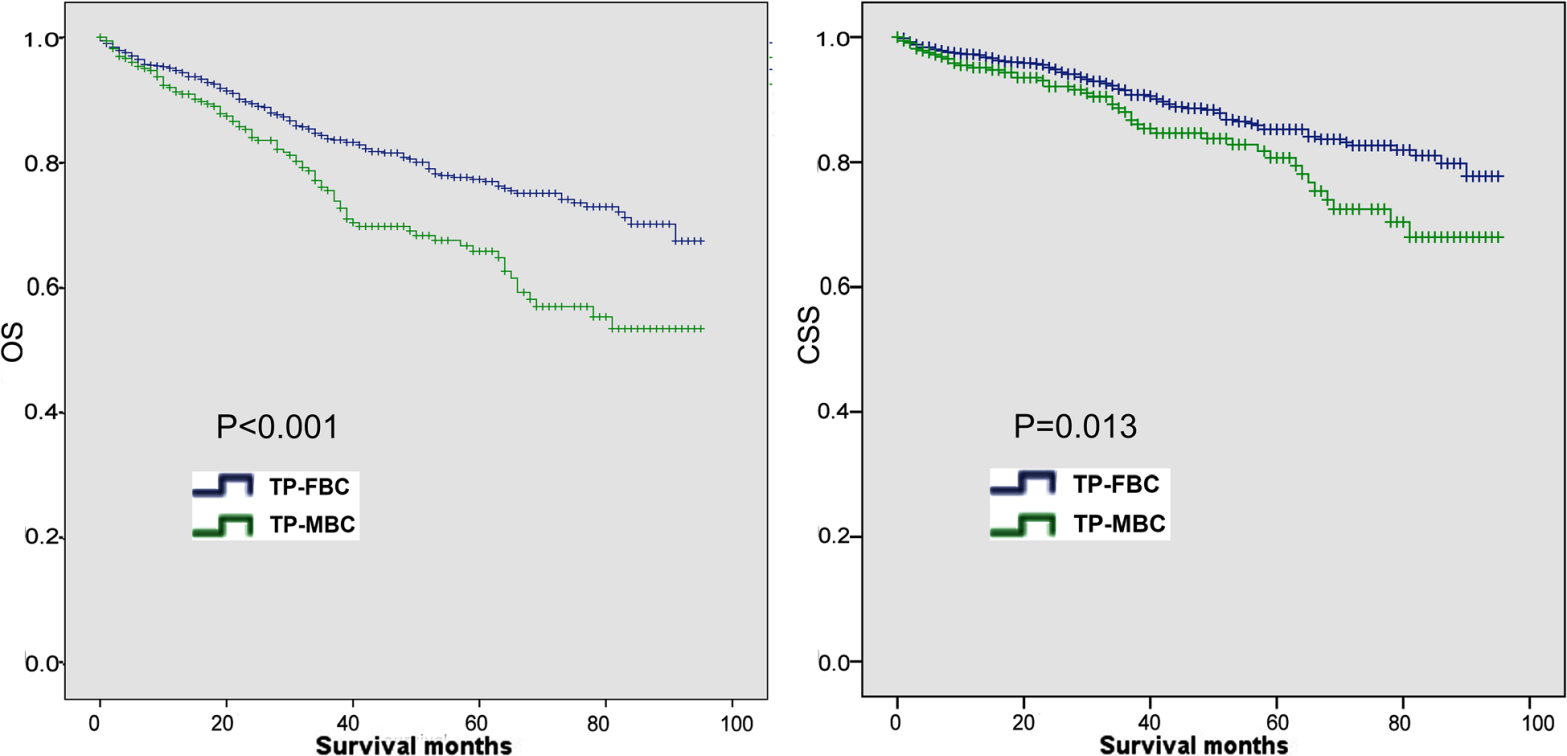
Comparison of OS and CSS in TP-MBC and TP-FBC after PSM

To reduce the bias between TP-FBC and TP-MBC, we used PSM to make each TP-MBC patient precisely matched with three TP-FBC patients. Thus, these two groups had similar clinic-pathological characteristics except sex (Supplementary Fig. S1; Supplementary Table S1). After PSM, there was still a significant difference (OS:*P* < 0.001; CSS:*P* = 0.013) between TP-FBC and TP-MBC in Kaplan–Meier analysis. TP-FBC had better OS/CSS than TP-MBC (Fig. 4).

### Mutation signatures

**Fig. 5:**
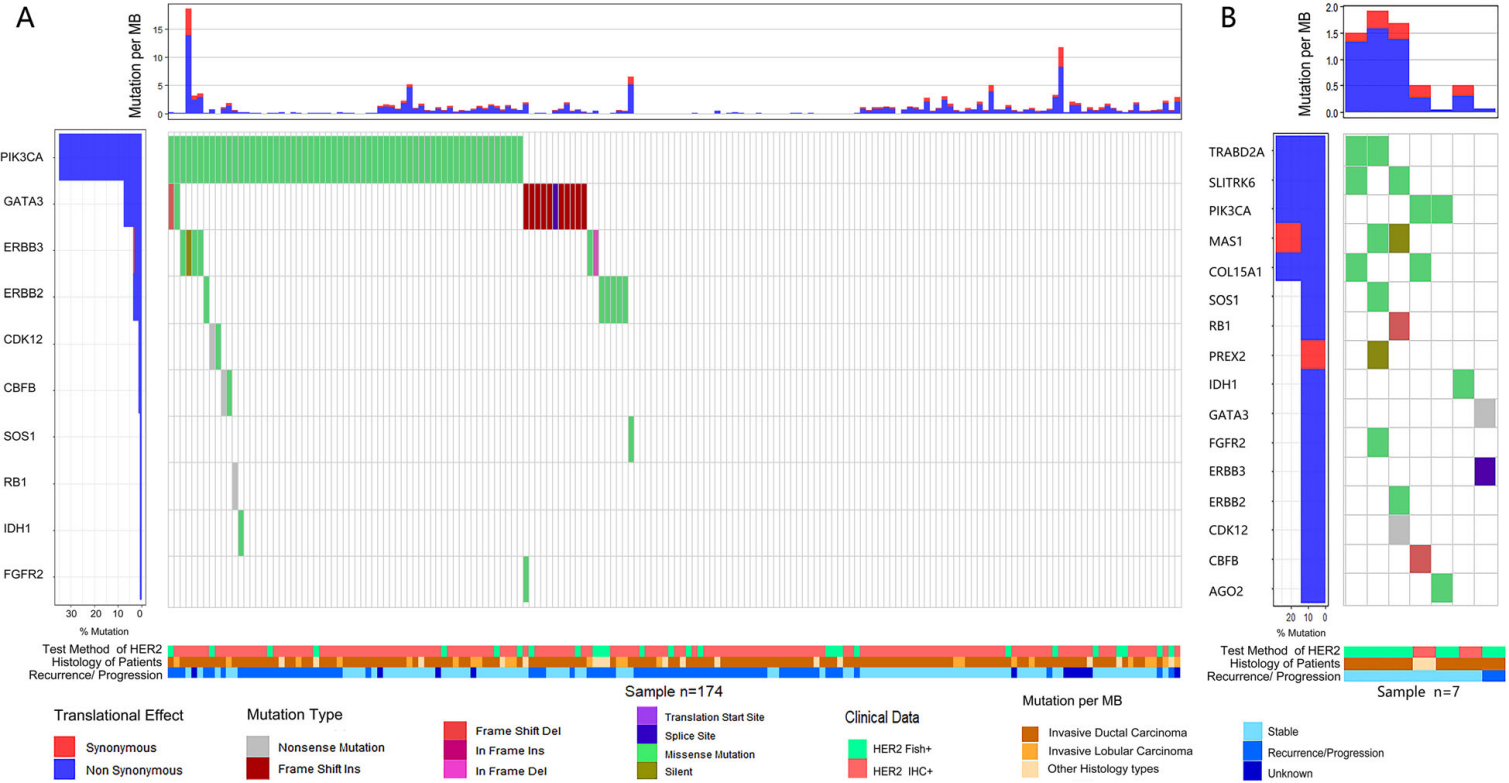
Genomic features of TP-MBC and TP-FBC

7 samples of TP-MBC and 174 samples of TP-FBC had mutation and clinical information. By analysing the mutation data, top 5 frequently mutated genes (28.6%) of TP-MBC were respectively TRABD2A, SLITRK6, PIK3CA, MAS1 and COL15A1. Among them, mutation rate of gene PIK3CA (35.1%) was higher in TP-FBC than that in male, while other four genes had no mutation in TP-FBC. Moreover, clinically relevant genes (including SOS1, RB1, PREX2, IDH1, GATA3, FGFR2, ERBB3, ERBB2, CDK12, CBFB, AGO2) showed mutation in TP-MBC (1/7, 14.2%). These genes showed infrequent mutation in TP-FBC (less than 10%) (Fig. 5).

## Discussion

Though MBC is a rare carcinoma worldwide, the delayed and challengeable treatment, as well as financial burden could not be ignored. Due to the uncertainty on the clinical outcome, genomic changes and the optimal treatment, it’s necessary to explore the difference between FBC and MBC. According to clinical routine, patients diagnosed with breast carcinoma would firstly confirm the molecular subtype. In MBC, the proportion of different molecular groups differ from female patients. As known to all, HR+ carcinoma occupied the vast majority of MBC, approximately 90% or more [18, 26]. A high level of HR expression in MBC could be summarized as follows: MBC is similar to breast cancer in postmenopausal women. However, the percentage of HER-2 positive MBC is still unsure due to the paucity of data and related studies. There were some studies found no/less (about 10%) amplification of the HER-2 gene in MBC compared with FBC [14, 27, 28], while other studies drew the conclusion that the HER-2 activation in MBC is as common as in FBC [26]. In fact, there are quite a number of researches support that MBC is mainly composed of luminal A and luminal B, and part of the latter are HR+/HER-2+ MBC [18]. Triple-positive breast cancer is a representative and important part, with advantages to showing the differences between men and women. By limiting the involved patients as triple-positive patients, our study aims to indicate the characteristics and prognose of unique part in MBC.

TP-MBC accounted for about 5.2–11.7% of the total MBC patients. Compared with TP-FBC, TP-MBC were more likely to be older than 65 years, White/Black patients, ductal carcinoma, bigger tumor size and later clinical stage. At the same time, there was a different tendency of metastases pattern between TP-FBC and TP-MBC. TP-MBC was easier to have distant metastasis such as bone and/or lung, which could supplement and support what Chong Li et al. reported [29]. TP-MBC had a worse OS/CSS than TP-FBC in total TPBC patients and in Kaplan–Meier analyses adjusted by clinical stage. The multi-cox models of OS and CSS in TPBC all showed sex is a prognostic parameter. Last, 1:3 accurate matched analysis between TP-MBC and TP-FBC indicated male TPBC patients did have worse outcome than female ones after adjusted by age, ethnicity, histology type, TNM stage. In a word, our research is the first to explore clinical-pathological features of TP-MBC compared with TP-FBC, and determine the worse outcome of TP-MBC.

Large-population based studies reported the worse/similar prognosis of MBC compared with FBC [3, 5, 17, 20, 30]. The contradictory outcome suggested there are subgroups of MBC with different survival risks. As Limin Peng et al. showed, when compared HR+ MBC with HR+ FBC, significantly worse OS of MBC had been found in some tumor stages but not all stages [22]. The phenomenon might be due to that HR+ MBC is a hybrid of multiple subgroups. It is interesting that patients have both activation of HR pathway and HER-2 pathway are originally thought to be relatively high-risk. Our data demonstrates that being male itself is the risk factor of TPBC’s OS and CSS. The finding is held up even after matching other clinical-pathological features. For these high-risk TP-MBC patients, the optimal treatment is not yet known and the enhancement of treatment is suggested in our conclusions.

The unfulfillment of adjuvant therapy (endocrine therapy, radiation therapy) [19, 31], the higher possibilities of relapse than FBC [20], the insufficient given of anti HER-2 drugs [10, 31] and a higher risk of contralateral tumors and second primary tumor [12] were reported to be possible reasons of worse outcome in MBC than FBC. Current management of MBC is mainly extrapolated from pre-clinical studies and clinical practice of FBC [2, 5]. Despite the lack of direct evidence, strategy of dual-blockade is still necessary for TP-MBC. Due to the survival advantage of tamoxifen in MBC compared with aromatase inhibitor [32], tamoxifen as a cornerstone of hormonal therapy should be given to TP-MBC. Other regimens of endocrine therapy on MBC still need more data, including fulvestrant. There is no data supporting that response to trastuzumab therapy is different for HER2-positive disease in men, so it is of importance to use trastuzumab in HER-2+ MBC. Since pertuzumab and trastuzumab can act synergistically, combination therapy of these two drugs may be proposed for trastuzumab-resistant MBC patients. In addition, Lapatinib may be a promising agent due to the effect on EGFR pathway. On the other hand, the use of CDK4/6 inhibitor in HR+/Her-2- MBC also has been granted approval by FDA, thus CDK4/6 inhibitor is also a promising drug in TP-MBC.

There are also some interesting findings on molecular profiling of TP-MBC. In our analyses for the somatic mutation data of TPBC, genes with high mutation rates in TP-FBC were different from mutated genes in male, like PIK3CA, TRABD2A, SLITRK6, MAS1, COL15A1 and key oncogenes like RB1, CDK12, ERBB2, ERBB3. Due to the number of TP-FBC is too small, we could not draw a clear conclusion. Jiang reported that TPBC was made up of several heterogeneous subgroups, and our results revealed sex is a key factor [33]. Some research also reported that androgen receptor (AR) plays a more prominent role in MBC than FBC [34, 35], thus development on AR inhibitor brings hope to these MBC patients [18, 36–38]. However, our study had not find AR mutation.

In our study, there are also some limitations. Our results are based only on data from the United States, and there might be differences among races. On the other hand, our genomic analyses based on a finite number of samples, which still need more clinical samples and sequenced data.

## Conclusion

Overall, our research is the first to demonstrate that TP-MBC is a more aggressive disease than TP-FBC. TP-MBC were easier found to be Black, and they tended to have bigger tumor size, later clinical stage and more distant metastasis to bone and/or lung than TP-FBC. Survival outcomes of these patients were worse than TP- FBC, even if we adjusted age, stage, race histology types in PSM analysis and multivariate analysis. At the same time, TP-MBC had some different mutated genes compared with TP-FBC, which might result in poor prognosis. It is important for clinicians to recognize the different characteristics and outcomes when they treat these patients. There is a great need for additional biomarkers, potential mechanisms and optional strategies to identify and treat these high-risk male patients.

## Supplementary Information

**Additional file 1.**

**Additional file 2.**

## Data Availability

Data in this manuscript is available in SEER database and CbioPortal online database.

## Abbreviations

ER: Estrogen receptor
PR: Progesterone receptor
HR: Hormone receptor
SEER: The surveillance, epidemiology, and end results
TP-MBC: Triple-positive male breast cancer
TP-FBC: Triple-positive female breast cancer patients
PSM: Propensity score matched
TCGA: The cancer genome atlas
MSK: Memorial sloan kettering
CSS: Cancer-specific survival
OS: Overall survival
HER-2: Human epidermal growth factor receptor
IHC: Immunohistochemistry
FISH: Fluorescence in situ hybridization
