# Supplementary figures and images for: Poor prognosis of male triple-positive breast Cancer patients: a propensity score matched SEER analysis and molecular portraits
Source: BMC Cancer. 2021 May 8;21:523. doi: 10.1186/s12885-021-08267-9 (PMC8106220; doi:10.1186/s12885-021-08267-9)

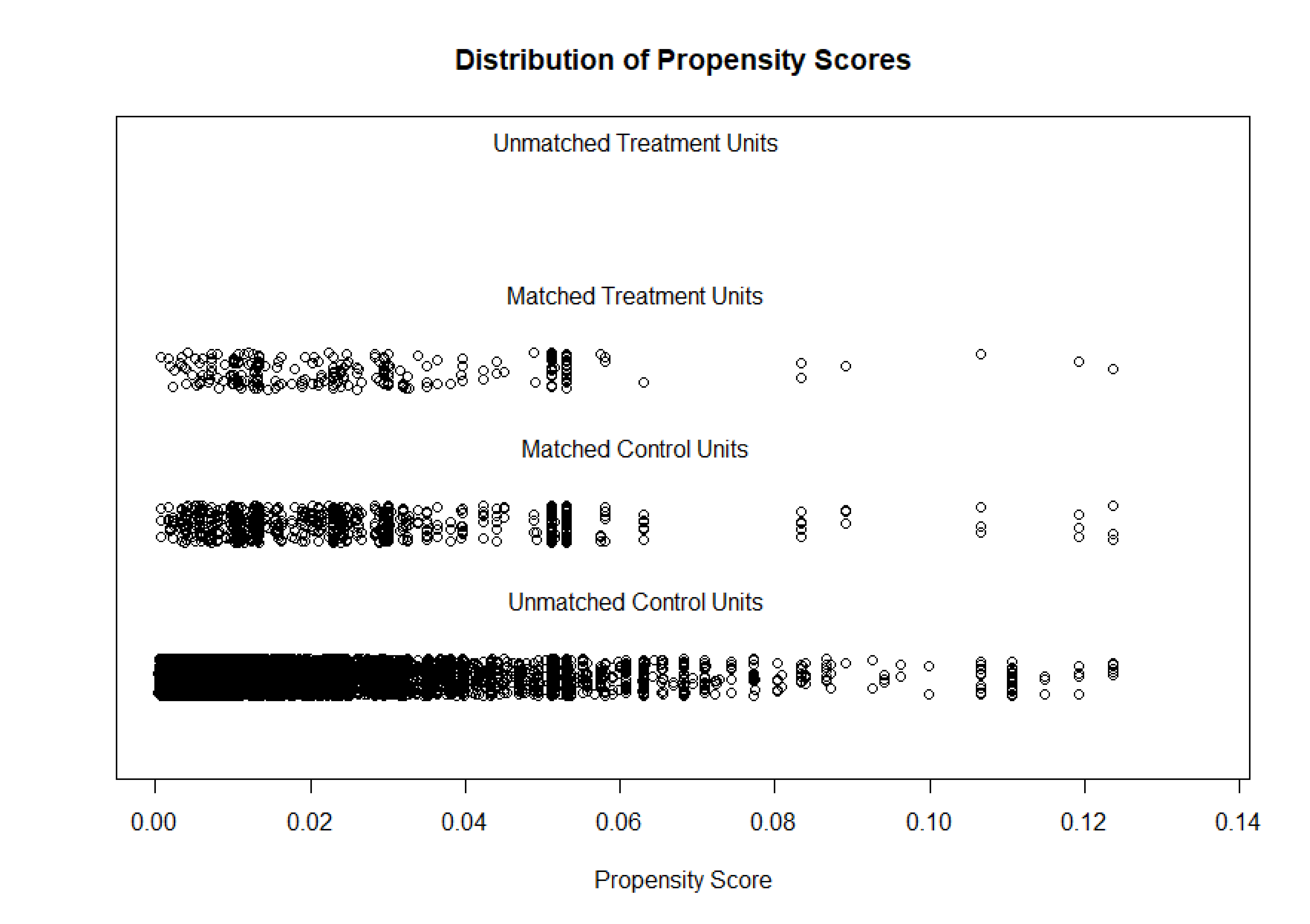

Supplement: Supplementary file 1 — Additional file 1. [file 12885_2021_8267_MOESM1_ESM.tif]
