# Supplementary material for: Poor prognosis of male triple-positive breast Cancer patients: a propensity score matched SEER analysis and molecular portraits
Source: BMC Cancer. 2021 May 8;21:523. doi: 10.1186/s12885-021-08267-9 (PMC8106220; doi:10.1186/s12885-021-08267-9)
Supplement: Supplementary file 2 — Additional file 2. [file 12885_2021_8267_MOESM2_ESM.docx]

Supplemental Table 1. Clinical pathological characteristics of TP-MBC compared with TP-FBC after PSM.

|  | TP-MBC (n=336) | TP-FBC (n=1008) | P-value |
| --- | --- | --- | --- |
| Age |  |  | 0.983 |
| ≤50 | 46(13.7) | 142(14.1) |  |
| 50＜ ≤65 | 132(39.3) | 395(39.2) |  |
| >65 | 158(47.0) | 471(46.7) |  |
| Race |  |  | 0.728 |
| White | 266(79.2) | 788(78.2) |  |
| Black | 51(15.2) | 146(14.5) |  |
| A/PI | 18(5.3) | 68(6.7) |  |
| Other | 1(0.3) | 6(0.6) |  |
| Histology |  |  | 0.573 |
| Ductal | 308(91.7) | 932(92.4) |  |
| Lobular and Mix | 13(3.8) | 43(4.3) |  |
| Other | 15(4.5) | 33(3.3) |  |
| Grade |  |  | 0.86 |
| I-II | 149(44.3) | 452(44.9) |  |
| III-IV | 171(50.9) | 515(51.1) |  |
| Unknown | 16(4.8) | 41(4.1) |  |
| Stage |  |  | 0.939 |
| I | 79(23.5) | 248(24.6) |  |
| II | 147(43.7) | 437(43.4) |  |
| III | 60(17.9) | 190(18.8) |  |
| IV | 37(11.0) | 98(9.7) |  |
| Unknown | 13(3.9) | 35(3.5) |  |
| T |  |  | 0.993 |
| T0-1 | 106(31.5) | 316(31.3) |  |
| T2 | 160(47.6) | 477(47.3) |  |
| T3 | 11(3.3) | 38(3.8) |  |
| T4 | 46(13.7) | 141(14.0) |  |
| Unknown | 13(3.9) | 36(3.6) |  |
| N |  |  | 0.995 |
| N0 | 160(47.6) | 481(47.7) |  |
| N1 | 118(35.1) | 355(35.2) |  |
| N2 | 29(8.6) | 90(8.9) |  |
| N3 | 20(6.0) | 54(5.4) |  |
| unknown | 9(2.7) | 28(2.8) |  |
| M |  |  | 0.771 |
| M0 | 297(88.4) | 905(89.8) |  |
| M1 | 37(11.0) | 98(9.7) |  |
| Unknown | 2(0.6) | 5(0.5) |  |
| Surgery |  |  | 0.455 |
| Yes | 285(84.8) | 872(86.5) |  |
| No | 45(13.4) | 126(12.5) |  |
| Unknown | 6(1.8) | 10(1.0) |  |
